# Profit Analysis of Papaya Crops under Greenhouses as an Alternative to Traditional Intensive Horticulture in Southeast Spain

**DOI:** 10.3390/ijerph16162908

**Published:** 2019-08-14

**Authors:** Mireille N. Honoré, Luis J. Belmonte-Ureña, Asensio Navarro-Velasco, Francisco Camacho-Ferre

**Affiliations:** 1 CIAIMBITAL Research Center, Agrifood International Excellence Campus, University of Almería, Carretera Sacramento s/n, 04120 Almería, Spain; 2 Department of Economics and Business, University of Almería, Carretera Sacramento s/n, 04120 Almería, Spain; 3 Vitalplant Nursery-Paraje Balsaseca, San Isidro-Níjar, 04117 Almería, Spain

**Keywords:** *Carica papaya*, alternative crop, transplant of sex-identified plants, productive structure for intensive crops

## Abstract

The high-yield agricultural model in Almería is based on eight different crops. Having led fruit and vegetable exports in Spain for more than 50 years, a decrease in melon and watermelon growing areas in Almería caused a change in supply that affected the model’s profit. Papaya cultivation could reactivate the profit of the agricultural model in Almería and also improve the available product range. The papaya crop needs greenhouse infrastructures high enough to contain the growth and size of the plants during a cycle crop, which is possible in most of the greenhouses of the Horticultural production model of Almería. The papaya harvests obtained in the region meet the quality requirements demanded by European markets. Furthermore, yields obtained are equal or higher than yields obtained by other producing countries. This crop improves profit compared with the profit obtained from the rotation of other horticultural crops that have been traditionally grown in the region.

## 1. Introduction

In the last 50 years, the province of Almería, in southeast Spain, has experienced a large economic transformation. The development of innovative and intensive agriculture has generated significant economic growth in the region with a positive impact on the welfare of families who live there [1]. This growth happened even though the region did not follow a proposal in European rural development guidelines to adopt an economic diversification strategy [2]. The productive diversification strategy is valid and advisable for developing countries, although it cannot be extrapolated to cover the whole of an increasingly liberalized world [3]. The agricultural sector in Almería has invested in new greenhouse architectures and technology to improve climate management and hydric resources. Moreover, product specialization has generated a local production system incorporating cooperation mechanisms that share innovative experiences and specialist knowledge.

The product specialization in Almería, sometimes called a high-yield agricultural model, is based on eight crops grown under plastic greenhouses: tomato, pepper, cucumber, courgette, aubergine, green bean, melon, and watermelon. Currently, such model is found in a productive maturity stage after developing a powerful commercialization channel. Increasing competition with third world countries is met through the reduction of production costs—mainly reducing labor and transportation costs [4,5]. Other costs, derived from crop insurance and greenhouse structures, are increasingly common among fixed costs, of a financial nature, on agricultural farms [6,7].

Production costs can be very variable among different production systems, due to the great differences in the technological levels and equipments used in each case. There are differences between different agrosystems due to the technological level of greenhouse cultivation, the contributions of energy, and inputs that they require [8]. However, one of the main components of the costs is the amortization of the investment in the structure of the greenhouse, in the equipment of fertigation, and in the climatic control. Therefore, as described Testa et al. [9], in the Mediterranean basin most of the greenhouses are built with low cost and technology in comparison to the greenhouses of Northern Europe [10,11], having plastic coverings and few climate control systems [12,13]. Thanks to the mild winters and the high solar hours, the structures do not need more investments to ensure a very aceptable quality crop. Genetic improvements of vegetables contribute to producing fruits that have a longer post-harvest life [14,15].

The adaptation process of the agricultural sector in Almería is demonstrated by the reorganization of the area grown, as shown in Table 1.

Considering the data obtained from the previous table and the cartographic base of greenhouses in Almería [16], three important conclusions are obtained:

1. The soil occupancy rate from 2009 to 2018 varied from 1.38 to 1.59, which means that the area used for short cycles is greater than that used for long cycles. Soil occupancy is the rate between the addition of areas occupied by the different crops developed in a season by the soil area available for the greenhouse. When a farmer cultivates only a crop in the greenhouse along the season in a long cycle, this rate will be equal to 1. When the farmer cultivates two short cycles in the same greenhouse (one in autumn-winter and a second in spring-summer) this rate will be equal to 2. An increase in the soil occupancy rate shows an augmentation of the use of short cycles in the greenhouses. This is a dimensionless number.

2. In one decade, the area used for greenhouses has increased by 3123 hectares.

3. The area used for each crop has changed. There was a decrease of 2157 and 411 hectares for melon and green bean, respectively. Meanwhile, the area used for the other crops has grown, mainly watermelon (4644 ha), courgette (3143 ha), and pepper (2676 ha).

**Figure 1:**
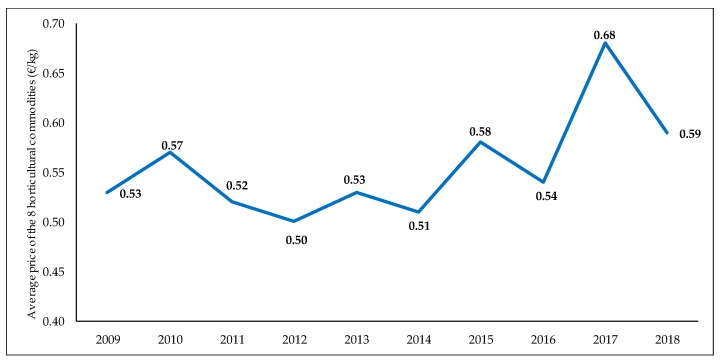
Evolution of the average price of the eight horticultural commodities grown under plastic greenhouses (tomato, pepper, cucumber, courgette, aubergine, green bean, melon, and watermelon) produced in the province of Almería (Spain) from 2009 to 2018, in €·kg^−1^ [17]. Source: Own elaboration made with data provided by the Provincial Delegation of the Regional Department of Agriculture of the Andalusian Regional Government in Almería (Spain) (2009 to 2018) [17].

With respect to commercialization, farmers maintained revenues over many years, mainly due to the stability of the average prices of the vegetable commodities (see Figure 1 and Table 2) produced in Almería, all mentioned in Table 1. However, as production costs increased year after year, the margins fell for units of product sold.

The data of Figure 1 are obtained from Table 1 and Table 2, calculating the weighted mean with the areas of each crop.

Two analyses can be made considering this price stability situation. On one hand, supply must decrease to meet a stable demand and, on the other hand, the product range that this model offers must increase to prevent loss of competitiveness within the sector.

Within this context, in order to expand the range of agricultural commodities offered by the province of Almería, we thought about working with an exotic species for the European continent such as papaya, obtaining production data and quality of the same as well as economic results of the sales and expenses incurred for production. The reasons for selecting the papaya crop in our study are twofold. First of all, in Europe, papaya consumption is growing due to its nutritional properties and high medicinal value [18,19]. Furthermore, the proximity of consumer markets to Southeast Spain allows fruits to be harvested at a more advanced stage of maturity compared to exporting countries from outside Europe, a timeline which improves the quality of the papaya.

Papaya (*Carica papaya* L) comes from southern Mexico and Nicaragua [20] and is a widely produced crop in the tropics. Until recently, the cultivation of this plant was carried out in tropical or subtropical regions. Nowadays, papaya is being cultivated in new regions using climatic protection techniques. In the province of Almería, some trials made on a very small scale by producers of the region have confirmed that this type of crop is well adapted to the agronomic conditions and production infrastructure found in southeast Spain. Additionally, there is an existing horticultural commercial network that provides a business infrastructure. The fruit were well accepted by the consumers. Additionally, in the economic situation of the province of Almería where there is a loss of competitiveness within the sector, we have observed that the papaya fruit is highly demanded by the European northern countries and that the average price is higher than the eight horticultural commodities cultivated under greenhouses.

In response to consumer demand we need to consider how product range can be expanded using the Protected Horticulture model from Almería. The trading sector has an agile commerce channel to sell the horticultural products, which has developed over more than 40 years, with an annual turnover of 3.5 million tonnes of fruit and vegetables for national and international markets. Within this framework and taking into account the special agro-environmental conditions and experience in the horticultural sector of Almería, the usual range of fruit and vegetables grown in this region could be extended, introducing a new crop such as papaya.

In the last decade, the demand for tropical fruits has grown at a constant and sustained rate. Papaya is the third most consumed tropical fruit, after mango and pineapple, and production reached 13,169,443 t in 2017 [21]. Although the increase in the papaya supply mostly derives from Indian production, this fruit has become a good source of revenue for many Asian and Latin American countries too.

In the last five-year period, over 50% of papaya production was distributed to the United States of America. In Europe, papaya consumption is consolidating due to the nutritional and high medicinal properties of the fruit [18,19]. The biggest importers of papaya in Europe are the United Kingdom, Holland, and Germany [22].

The Canary Islands (Spain is Europe’s largest papaya producer with cultivation on 350 ha [23]. This cultivation area is expanding, meanwhile the area used for growing tomatoes is decreasing. Papaya is also grown in Continental Europe. In 2012, cultivation first began in the province of Almería on 6000 m^2^. The production obtained was marketed locally as “papaya of Almería”. Early on, studies took place to understand both the adaptation of this crop to the southeast Spain, greenhouse production system, and how different cultivation practices could improve yield.

The aim of this article is to present the advantages of incorporating a subtropical crop like papaya and increase the range of products offered by the Almería agricultural sector, which tends to concentrate the supply on only eight horticultural commodities. Papaya cultivation was selected for several reasons. First, there is an important European demand for this crop, and India is currently the first supplier. Another reason for this choice is that Papaya can be cultivated under greenhouses with the productive conditions of the Spanish southeast. For both reasons, a field study was carried out in Almería to assess the quality of a Papaya cultivation grown under a naturally ventilated greenhouse and to estimate the net profit before taxes that the papaya crop would generate, over a period of 2.5 years (30 months). A comparison is made with the most common horticultural crop combinations in South Eastern Spain, crops that have been grown in the province of Almería for four decades. Finally, from the point of view of the distribution, this crop has been incorporated into the offer of several suppliers in the region, even with scarce importance due to the reduced cultivated area now in the province.

## 2. Materials and Methods

An experiment was conducted to obtain the product. From this experiment, production and quality (agronomic) data were obtained alongside economic data concerning when papaya was put on the market. The greenhouse where it was planted has an area of 1800 m^2^, and this area corresponds with the area of the greenhouse located in the experimental plot of the University of Almería.

### 2.1. Location of the Experiment

The experiment was conducted at the research station “Catedrático Eduardo Fernández”, which belongs to the Foundation of the University of Almería-Anecoop. It is located in a place called “Los Goterones” in Almería, 36°51′ North latitude and 2°17′ West Longitude, at 90 m above sea level.

### 2.2. Greenhouse Infrastructure

**Figure 2:**
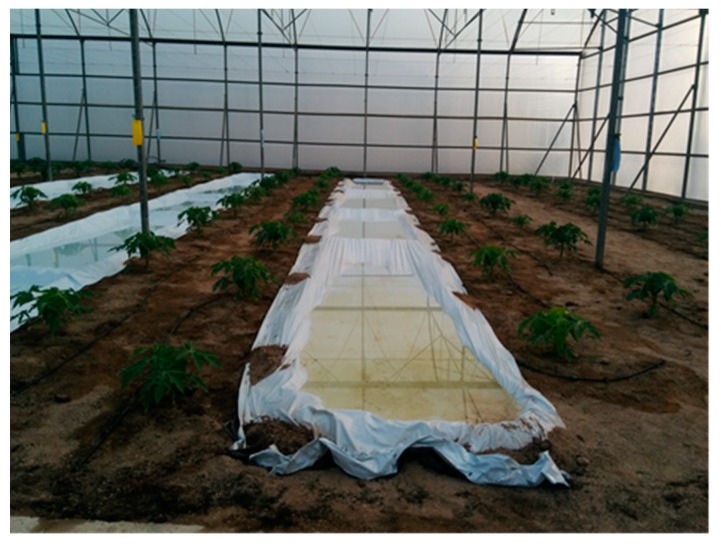
Plastic mulching of part of the floor surface for the incorporation of humidity in the environment.

The plants were transplanted in a “multi-tunnel” greenhouse with an area of 1800 m^2^, a gutter height of 4.50 m, and a ridge height of 5.70 m. The covering was made from three layers of plastic film (low-density polyethylene, 200 microns thick). Due to the climatology of the region and the needs of the papaya plant to be within the above parameters, from June to September three side walls were opened and the East oriented side wall was closed; wind is warm in this orientation, so there is an excessive decrease in the relative humidity in the interior of the greenhouse. To avoid this decrease in relative humidity, white plastic mulch film was placed on the soil, in alternate lines, forming small reservoirs which were filled with water to produce evaporation (Figure 2). In winter, this mulch film was not filled with water and alternate lines were mulched with black plastic film because covering the whole soil surface decreases relative humidity.

The soil used was enhanced to place its quantitative parameters of nutrients and organic matter within the range of fertile soils. The soil was protected with a sand covering, which is typical in this region, as described by Camacho and Fernández [24]. During the past 13 years, tomato, pepper, courgette, and cucumber were grown in this soil.

Irrigation was carried out through a localized irrigation system of high frequency (drip irrigation) using discharge drippers 3 L·h^−1^ with a density of 1.6 drippers/m^2^.

### 2.3. Plant Material

The vegetal material used was Intenzza papaya seeds, belonging to the “Semillas del Caribe” company (Jalisco, Mexico) [25]. Out of the current varieties grown under greenhouse in Continental Europe, this specific variety was chosen because it is the leading variety. The vegetal material was prepared in the nursery to conduct the experiment in the plot described above.

The characteristics of the planted papaya were a sex-identified Intenzza Papaya with a rootball (pot) of 2 L and an approximate plant height of 60 cm. The sowing date was 10 October 2015 and the early sex-identification was made on 20 November 2015, 40 days after sowing (das).

The transplant to the final field was made on 23 March 2016. A total of 30 months (February 2016 to July 2018) is the period during which the analysis of the data of this experiment was made. The papaya cultivation in this trial started on March 2016 (transplanting date). Preparation of the soil was in February 2016 and during July of 2018, when the plants were taken off.

The distribution of plants was made in paired and staggered rows with a distance of 2.20 m (corridors) between paired rows, 1.00 m between lines and 2.00 m between plants. Under these distributions, there was a density of 2700 plants/ha. The total area of the experiment was 1650 m^2^ used for growing, the rest of covered area was used as walkways.

### 2.4. Fertilization and Crop Protection

The soil had a clay loam texture with 0.45% of organic matter and pH and electrical conductivity (EC) values, in the saturation extract, of 7.80 and 4.65 dS·m^−1^ respectively.

The water used had an EC of 1.4 dS·m^−1^ and a pH of 7.13. Nutrients were applied by drip irrigation.

**Figure 3:**
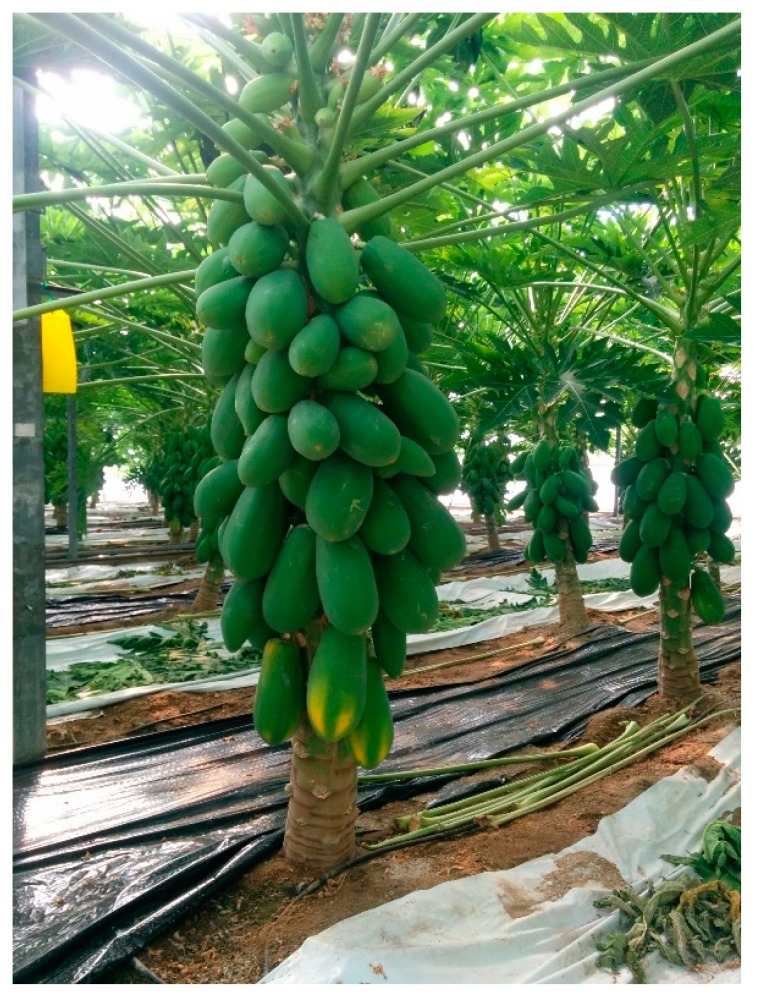
Papaya plant under greenhouse with fruits close to harvest.

One week after transplanting, the addition of fertilizer began. Additionally, the EC of irrigation water increased by 0.5 dS·m^−1^ until it progressively reached 2 dS m^−1^, the maximum amount of EC reached by addition of nutrients, for plants developed from the beginning to the end of the harvest (Figure 3).

Crop pests and diseases were controlled with biological control with releases of beneficial insects, or natural pesticides. *Tetranichus urticae* were controlled with *Phytoseiulus persimilis* and *Amblyseius californicus* releases, in addition to the occasional appearance of *Stethorus punctillum*. The small aphids *Aphis gossypii* were controlled with preventive *Lysiphlebus testaceipes* and *Aphidius colemani* releases on cereal banker plants, which were infected by us with a specific aphid, *Rhopalosiphum padi*. In winter, *Botrytis cinerea* appeared in small areas injuring the stem, which caused the fall or removal of the lower petioles. It was controlled by applying Samurai^®^-Nutricrop paste (Almería, Spain) (a product made from natural clays that isolates the internal tissues of the plant from the outside environment) locally and on the injured stem. At the end of winter, *Oidium caricae* also appeared on small areas and it was controlled by applying Ospo V^®^-Agrotecnología sulphur (Orihuela, Spain) (a product made from vegetal extracts, flavonoids, alkaloids, phenols, macro and microelements, polysaccharides and microorganism extracts) and Larekigreens^®^, Biofungitek, (Derio, Spain) (a product made from potassium carbonate and vegetal extracts).

### 2.5. Harvest Time

The fruit harvest was carried out from October 2016 to the beginning of August 2017, and it began 193 days after transplanting (dat), first season. Then it began at the end of August 2017 until the middle of June 2018 (second season). Within the mentioned periods, the harvest was carried out every week in color stage 2 to 3 using the color stage values of Santamaría-Basulto et al. [26]. The maturity stage 2 corresponds with yellow coloring fruit between 25% and 33%. The maturity stage 3 corresponds with yellow coloring fruit between 33% and 40%. In the spring-summer weeks, the harvest was only carried out at maturity stage 2. The harvest was carried out one day per week in winter and four days per week in spring-summer.

The fruits were weighted with a BBA422-60LA BASIC (Mettler Toledo, L’Hospitalet de Llobregat, Spain) scale with a maximum capacity of 60 kg and ± 1 g sensitivity.

### 2.6. Comparison of Economic Data between the Papaya Cultivation and Crops currently Grown in the High-Yield Agricultural Intensive Model in Almería

The economic comparison was made under the principles of maximization of benefits by farmers. Hence, to evaluate the opportunity of the different crop alternatives, the net profit before taxes (NPbt) has been used for each possible crop combination [27,28,29]. NPbt was calculated as a difference between the total annual sales revenue (TAR) and the total costs (TC) incurred in each campaign.

NPbt = TAR − TC

With NPbt: Net Profit before taxes; TAR: Total Annual Revenue; TC: Total costs.

The structure of revenues and expenditure which was followed to conduct this research, was agreed by the “Experimental Plot of the Foundation University of Almería-Anecoop”, where the experiment was conducted. Additionally, the structure obtained by Toresano and Camacho for Agroseguros, S.A.-Spain [30] was followed, data not published corresponding with the Provision of Services PS20120000000000184 of the Research Results Transfer Office (OTRI) of the University of Almería.

To make a comparison between the crops, five different agronomic alternatives were taken as a reference from the species with which the model works (four cucurbits, three solanaceous, and one leguminous plants). In this sense, a different crop was planted after each cycle, except for the case of long-cycle tomato. Therefore, the first four alternatives were formed by crops developed in short cycles, every six months (two harvest per year), and the fifth alternative began with a courgette crop, short cycle (6 months), and when it finished, two tomato harvests followed it, long cycle (12 months), until total period of 30 months was completed.

These alternative crops are usual in the intensive agriculture model in the Spanish southeast [8].

To make an economic comparison, the horticultural alternatives studied against the new papaya crop were:

Horticultural 1: watermelon (2016) + tomato (2016) + courgette (2017) + pepper (2017) + watermelon (2018).

Horticultural 2: tomato (2016) + cucumber (2016) + aubergine (2017) + green bean (2017) + melon (2018).

Horticultural 3: melon (2016) + pepper (2016) + watermelon (2017) + tomato (2017) + melon (2018).

Horticultural 4: courgette (2016) + aubergine (2016) + melon (2017) + pepper (2017) + watermelon (2018).

Horticultural 5: Courgette (2016) + tomato (2016–2017) + tomato (2017–2018).

The technical characteristics of the productive infrastructures, to obtain economic data with respect to investment cost and its corresponding depreciations, are as follows:

Papaya: Multitunnel greenhouse described above in Section 2.2.

Long-cycle horticultural crops: Almería type “raspa y amagado” greenhouse with 6.00 m ridge height, 5.00 m gutter height (amagado), and 4.70 m wall height [8].

Short cycle horticultural crops: Almería type “raspa y amagado” greenhouse, with 4.50 m ridge height, 3.50 m gutter height (amagado), and 3.00 m wall height. These are the dimensions of the “fashionable greenhouse” obtained by Valera et. al. [8]

The rest of infrastructures of production, climate control, and irrigation systems were the same in the different cases analyzed. The energy costs in the papaya cultivation and in the horticultural alternatives were the same in this study. The greenhouses were naturally ventilated without any heating system.

The amount of the total revenues from papaya cultivation was obtained from the real value in euros of the production which was sold during the corresponding field trial. In the case of horticultural alternatives (Horticultural 1 to Horticultural 5), the value of the total revenues was calculated by multiplying the average yield (kg/m^2^) of each crop by the corresponding average campaign price (€/kg) (see Table 2 and Appendix A, Table A1) [17].

With respect to current expenditure, technical assessment was the same for the eight crops studied. Soil preparation was adjusted depending on the cycle length, 6, 12, or 30 months. The covering and structure depend on the type of greenhouse used and the size of the plastic covering, which also depends on the greenhouse size and the difference of labour costs to replace the greenhouse plastic which, in turn, and also depends on the structure and height of the greenhouse covering. For seeds and seedling production, the data were obtained according to the costs of each year.

In the case of water, fertilizers, phytosanitary products, labor costs, stakes, auxiliary insects, and management of crop residues, the guidelines marked individually for each crop and cycle by Toresano and Camacho [30] were followed. It is important to highlight the economic percentage weight that labor costs have within the current costs, once the work units were assessed (Table 3).

## 3. Results and Discussion

Based on the obtained yield, the harvest of papaya (after assessing the yield parameters, average fruit weight, and soluble solids in °Brix) is appropriate to put papaya on the European market, since they have similar characteristics to those that are being imported to Europe from other continents.

### 3.1. Total Yield of Papaya

In the first season, 81 harvests were carried out between weeks 41 in 2016 and 31 in 2017. In the second season, 68 harvests were carried out between weeks 35 in 2017 and 25 in 2018. The total yield was 27.63 kg·m^−2^ (see Appendix A, Table A2).

The results obtained in this experiment of 276.3 t·ha^−1^, are higher than those obtained in studies from Costa Rica by Guzmán Díaz [31], which varied between 40 and 70 t·ha^−1^, and Jiménez Díaz [32], who obtained 89 t·ha^−1^ in a three-year cycle. In Mexico, Escamilla et al. [33] conducted an experiment to study yield of cultivarMaradol papaya and obtained 27.94 t·ha^−1^. In India Singh et al. [34] reported yields between 65 and 93 t·ha^−1^. In other experiments in India, Jeyakumar et al. [35], reported yields between 139.3 and 184.9 t·ha^−1^. and Bhalerao et al. [36] obtained between 51.83 and 80.76 t·ha^−1^. Migliaccio et al. [37] carried out an experiment in Florida (USA) and obtained yields between 152 and 193 t·ha^−1^.

The difference in total production of papaya produced in Almería with respect to total production for other countries mentioned, is due to the production system. In effect, in Almería the papaya is cultivated under greenhouses while in the rest of the countries, this is an open field cultivation.

### 3.2. Average Fruit Weight (AFW) of Papaya

The fruits harvested in the second season weighed more than those obtained in the first season.

These data are consistent with those obtained by Pérez Hernández [23], in an experiment conducted with 10 different varieties, where the fruit harvested in the second season weighed more than those obtained in the first season.

The weight obtained in the fruits has an acceptable demand in the European market. The weight of the Intenzza fruit in this experiment had the size G (801–1100 g) and H (1101–1500 g), described in the International FAO (Food and Agriculture Organization of the United Nations) Standards for papaya cultivation [38].

### 3.3. Number of Fruits per Plant

The number of fruits per plant in the second season was lower. There is a relationship between fruit size and number of fruits per plant.

### 3.4. Total Soluble Solids

The analysis of total soluble solids was made for the °Brix average and for the maximum °Brix in fruits. The values obtained throughout the harvest time varied between 10.00 and 10.30 °Brix.

These values were similar to those obtained by Pérez Hernández [23] for the Intenzza variety grown in the Canary Islands. The values are also similar to the results obtained by Santamaría et al. [39] in Costa Rica. The data obtained in the experiment permitted the sale of papaya fruits in different European countries.

### 3.5. Comparative of Results: Horticultural Alternatives Versus Papaya Cultivation

After the analysis of the structure of revenues and costs of the main combinations of horticultural crops in the southeast of Spain, a comparison of the economic yields was made with those obtained by papaya cultivation as described above (see Table A2, Table A3, Table A4, Table A5, Table A6 and Table A7 in the Supplementary Material, Appendix A). In the case of papaya cultivation, the real costs and revenues were taken into account, whereas in the case of the different horticultural alternatives, the production data and prices used were published by official bodies [17,40]. Furthermore, the detail of horticultural costs, attributable to each alternative, comes from the cost analysis updated by Toresano and Camacho for Agroseguros, S.A.-Spain [30]. The results of the comparison of papaya crop with five crops that are usually cultivated in the Province of Almería are detailed and represented in Table A2, Table A3, Table A4, Table A5, Table A6 and Table A7 (see the Supplementary Material, Appendix A). The data of Table 4 are obtained from Table A2, Table A3, Table A4, Table A5, Table A6 and Table A7 and represent a resume of the comparison for the five crops.

Table 4 shows the profit and loss account of each of the alternatives that are compared, that is, the five horticultural combinations with papaya cultivation. In the case of papaya, the revenues that were calculated in the experiment were those received by the sale of this fruit. The data are obtained from Table A2 (see Supplementary Material, Appendix A). All the papaya fruits coming from the Experimental Plot of the Foundation University of Almería-Anecoop were put on the market through a company operating in the region, which is a specialist in introducing new fruits to European markets. This company grew 10 papaya hectares on its own plots of land and on other plots belonging to its associates. In all the cases, the comparisons were made for the same period, from February 2016 to July 2018.

**Figure 4:**
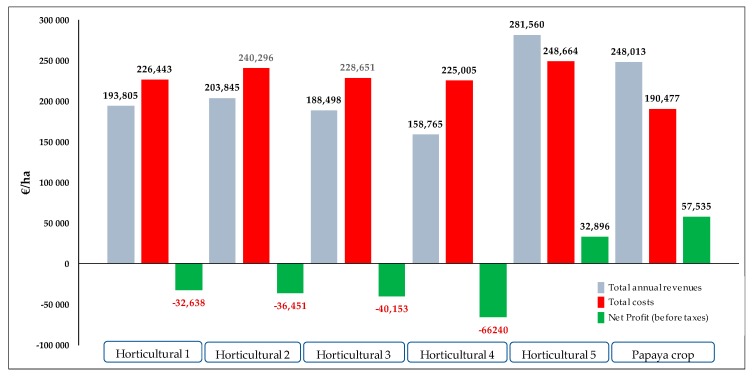
Comparison between the economic results (€/ha) of the proposed horticultural alternatives and the cultivation of the papaya in a 30-month cycle (2016–2018). Total annual revenues (█); Total costs (█); Net Profit (before taxes) (█). Own elaboration.

The data of total annual revenues for each cultivation in this table are provided in the Supplementary Material—Table A2, Table A3, Table A4, Table A5, Table A6 and Table A7 in Appendix A. To have a global visualization of the comparison between the five horticultural alternatives and papaya crop, the total annual revenues, the total costs, and the net profit (before taxes) are represented in Figure 4 for each case. The data were obtained from Table 4. Figure 4 shows the comparison between papaya cultivation in southeast Spain with the different proposed horticultural alternatives. In this figure, four out of the five combinations of horticultural crops generate losses within the period analyzed (February 2016 to July 2018) and only the combination called “Horticultural 5” gives profits.

**Figure 5:**
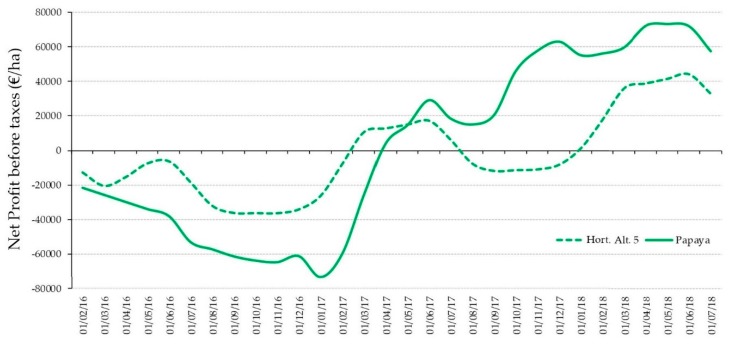
Monthly evolution of the accumulated Net Profit before taxes (NPbt) for Horticultural alternative 5 (**---**) versus papaya cultivation (**—**) (€/ha) during the trial period (February 2016 to July 2018). Own elaboration [17,30].

The question that needs to be asked is how can this horticultural production model be maintained, especially in the “Horticultural 1 to Horticultural 4” combinations? As certain studies had anticipated [41], the answer must be sought in the structure of the revenue statements of each farm and not from a business logic, profit maximization, but in a way to subsist in a mature sector. In this sense, there are two accounting entries that are not assessed in terms of opportunity costs by the owners of the farming business. These are labor costs and annual depreciation costs:

1. There are some studies that place labor costs between 25% and 40% of the annual costs of a farming business under greenhouses [42,43]. In this sense, the field study that was carried out by Valera et al. [8] showed that the owner of the farming business, together with their staff, is another worker. Therefore, when there are losses, as described in the combinations “Horticultural 1 to Horticultural 4”, the owner renounces his remuneration as owner of the means of production because they tend to reduce staff numbers and, together with their family, take roles which, during favorable economic conditions, would be filled with employed workers.

2. The annual depreciation cost of the greenhouse structure, the irrigation system and pools, represents the main part of the fixed costs of the farming business. According to Calatrava and Villa [44], the fixed costs of a greenhouse used for horticulture varies from 1 to 1.5 €/m^2^. This result is in accordance with our calculations per hectare and cycle (see “Total fixed costs (€)”, in Table A2, Table A3, Table A4, Table A5, Table A6 and Table A7, in Supplementary Material, Appendix A). In this case, the depreciation cost can reach 50% of the total fixed costs, from 4000 to 5000 Euros per hectare and cycle. In the case of producers with lower indebtedness and that have already paid for the farming business, they could have these amounts available because they would not be obliged to depreciate yearly. Considering the average area of the farming business in Almería, 23,508 m^2^ [45], the depreciation costs of the greenhouse structures, irrigation systems and pools, would be higher than the costs stated in this article. Furthermore, the level of subsidies that the Almería intensive horticulture receives is low in relation to its size [8] and as has already happened in other productive sectors of the province. In the foreseeable future the subsidies should be reduced [46].

3. Finally, we calculated the accumulated net profit before taxes for each comparison of the horticultural alternatives and papaya crop during the period of 30 months (February 2016 to July 2018), time used for the trial with the papaya crop. Figure 5 represents the visualization of the accumulated monthly Net Profit before taxes (NPbt) for the most profitable crop (Horticultural 5 and the papaya cultivation, see Table A2 and Table A7) during the period of the trial (30 months). In both cases, there was a positive net profit. Monthly, the calculated NPbt in each alternative is added the NPbt of the last month and cumulated, until the end of the period. The “Horticultural 5” contains a cycle of courgette cultivation and two cycles of tomato cultivation. Analyzing Figure 5, it can be highlighted that the farmers which choose the papaya cultivation should be conscious that they must have enough liquidity to maintain their business for 11 months (March 2017, in Figure 5). At this moment, the NPbt is positive. In the case of Horticultural 5, after 3 months (May 2016), the farmer can recover part of his investment, althought the cumulative NPbt is still negative. Most of the time, farmers prefer horticultural crops with short cycle, of 6 months, for which they begin to receive revenues for the sale of their products and to improve the cumulative NPbt, 40–45 days after sowing.

## 4. Conclusions

After analyzing the development of the areas and production of the main horticultural crops in southeast Spain, as well as the evolution of the average prices from 2009 to 2018, there are evident signs that the horticultural production model in Almería is at a mature stage. In addition to this, there is also an assessment of the alternative of papaya crops grown under greenhouses. After making a comparison of papaya crop with five crops that are usually cultivated in the Province of Almería, it can be observed that only one horticultural alternative gives positive results, from February 2016 to July 2018. For each one of those crops, the cultural system is very particular, and this is the reason why the comparison must be taken in the context of the province of Almería. For the cultivation of papaya, there was only one plantation (from February 2016 to July 2018, 30 months), and in the alternative five, three crops were planted (one cycle of courgette and two cycles of tomato), thus, to achieve a planting period similar to the papaya crop.

A detailed analysis of the profit and loss account for each of the horticultural alternatives allows a relationship to be drawn between the good results of the season with high sale prices, compared with the average of previous seasons in 2017, the year with best economic result for the horticultural production model in Almería. In 2017, an average price of 0.68 €·kg^−1^ was registered compared with the range from 0.50 to 0.59 €·kg^−1^ that was registered in the period 2009–2018. The producers of long-cycle tomatoes are the only ones who achieve reduction in their variable costs (in the phases from the “seed to the seedling in nursery” and the phase from “flowering to first harvesting season”), making the harvest period more continuous and, therefore, obtaining revenues from the sales of the horticultural commodities.

With the results of this work, it is demonstrated that papaya can be one of the crops for the Province of Almería to extend the range of products offered to the Northern European market. The papaya cultivation under greenhouses in this region is feasible, with higher yield obtained than in other countries, with local mature commercial channels which have the how-know to sell exotic commodities complying with the European marketing standards. The cultivation of this fruit under greenhouses in Almería can be a rentable and commercial activity for farmers.

## Figures and Tables

**Table 1:** Evolution of the wintering area by type of crop (2009–2018) in hectares.

Year	Tomato	Sweet Pepper	Cucumber	Aubergine	Courgette	Green Bean	Melon	Watermelon	Total Area Green-House
2009	10,147	7505	4430	1868	4717	921	4447	5216	39,251
2010	9939	7475	4498	1824	5020	776	4039	5516	39,087
2011	9050	7300	4550	1924	5265	680	3539	4916	37,224
2012	9124	7388	4535	2190	5789	1170	3740	5665	39,601
2013	10,358	8461	4920	2006	6448	1321	4200	6400	44,114
2014	11,206	9378	4839	1908	7219	1387	2591	7100	45,628
2015	10,345	9326	4979	2447	7477	1439	2946	8378	47,337
2016	10,940	9491	5026	2300	7630	1340	2467	8590	47,784
2017	10,220	10,310	4980	2150	7970	1030	2220	8940	47,820
2018	10,380	10,181	5099	2209	7860	510	2290	9860	48,389
Change (from 2009 to 2018)									
Abso-lute	233	2676	669	341	3143	−411	−2157	4644	9138
**%**	2.30	35.60	15.10	18.20	66.70	−44.60	−48.50	89.00	23.28

Source: Own elaboration made with data provided by the Provincial Delegation of the Regional Department of Agriculture of the Andalusian Regional Government in Almería (Spain).

**Table 2:** Average price of the eight horticultural commodities grown under plastic greenhouses (watermelon, melon, courgette, cucumber, aubergine, tomato, pepper, and green bean) produced in the province of Almería (Spain) from 2009 to 2018, in €·kg^−1^ [17].

Crop	2009	2010	2011	2012	2013	2014	2015	2016	2017	2018
Watermelon	0.242	0.249	0.332	0.250	0.263	0.278	0.372	0.318	0.327	0.407
Melon	0.336	0.398	0.376	0.395	0.381	0.367	0.451	0.470	0.448	0.535
Courgette	0.499	0.670	0.380	0.475	0.564	0.463	0.789	0.489	0.681	0.555
Cucumber	0.577	0.446	0.434	0.435	0.542	0.449	0.467	0.418	0.640	0.501
Aubergine	0.570	0.503	0.448	0.418	0.591	0.511	0.453	0.371	0.728	0.513
Tomato	0.560	0.646	0.511	0.571	0.525	0.538	0.559	0.527	0.714	0.621
Pepper	0.723	0.746	0.882	0.668	0.768	0.647	0.733	0.824	0.908	0.755
Green Bean	1.281	0.902	1.459	1.518	1.360	1.127	1.575	1.455	1.747	1.584
Average	0.599	0.570	0.603	0.591	0.624	0.548	0.675	0.609	0.774	0.684

Source: Own elaboration made with data provided by the Provincial Delegation of the Regional Department of Agriculture of the Andalusian Regional Government in Almería (Spain) (2009 to 2018) [17].

**Table 3:** Economic percentage weight of labor on current expenses of horticultural crops that occur in the southeast of Spain (Toresano and Camacho) [30].

Culture	Tomato	Sweet Pepper	Cucumber	Aubergine	Courgette	Green Bean	Melon	Watermelon
Percentage	52.66	47.32	45.40	52.07	86.44	42.00	22.33	21.19

**Table 4:** Structure of revenues and costs per hectare of five horticultural alternative crops versus papaya cultivation, analyzed from February 2016 to July 2018.

Comparison. Feb 2016 to July 2018	Horticultural Alternative 1	Horticultural Alternative 2	Horticultural Alternative 3	Horticultural Alternative 4	Horticultural Alternative 5	Papaya Cultivation
Total annual revenues (€)	193,805	203,845	188,498	158,765	281,560	248,013
Total variable cost (€)	177,390	191,244	179,599	175,953	194,971	135,618
Technical assessment (€)	772	772	772	772	772	772
Soil preparation (€)	19,458	19,458	19,458	19,458	11,575	3839
Covering and structure (€)	11,846	11,846	11,846	11,846	11,846	12,297
Seeds and seedling production (€)	17,983	22,588	25,630	18,741	14,689	13,500
Growing and development until 1st inflorescence (€)	41,947	35,401	34,958	43,591	32,898	33,131
Flowering periods until 1st harvesting season (€)	25,779	33,679	25,862	18,771	31,380	37,570
From the 1st harvesting season until the end of the cultivation (€)	59,605	67,499	61,073	62,772	91,811	34,510
Total fixed costs (€)	49,052	49,052	49,052	49,052	53,693	54,859
Soil maintenance (€)	5140	5140	5140	5140	5140	5125
Covering and structure (€)	10,287	10,287	10,287	10,287	10,287	10,257
Energy and fixed supplies (€)	4063	4063	4063	4063	4063	4051
Insurance, management, and financial services (€)	8947	8947	8947	8947	8947	8920
Equipment and irrigation system (€)	20,615	20,615	20,615	20,615	25,256	26,506
Total cost (€)	226,443	240,296	228,651	225,005	248,664	190,477
Net profit before taxes (€)	−32,638	−36,451	−40,153	−66,240	32,896	57,535

Source: Own elaboration from a field trial (papaya), Toresano and Camacho [30] and Annual Report of the Regional Department of Agriculture, Rural Development and Fisheries [17].

## Appendix A

**Table A1:** Area, production, and price of horticultural crops in years 2016–2018.

Crop	2016			2017			2018		
	Area (ha)	Production (t)	Price (€·kg^−1^)	Area (ha)	Production (t)	Price (€·kg^−1^)	Area (ha)	Production (t)	Price (€·kg^−1^)
Watermelon	8590	532,288	0.318	8940	558,223	0.327	9860	512,742	0.407
Melon	2467	96,417	0.470	2220	93,527	0.448	2290	91,656	0.535
Courgette	7630	434,195	0.489	7970	448,975	0.681	7860	456,045	0.555
Cucumber	5026	438,870	0.418	4980	422,214	0.640	5099	443,604	0.501
Aubergine	2300	184,161	0.371	2,150	168,046	0.728	2,209	181,130	0.513
Tomato	10,940	1,107,706	0.527	10,220	1,008,867	0.714	10,380	996,254	0.621
Sweet pepper	9491	665,922	0.824	10,310	694,402	0.908	10,181	732,989	0.755
Green bean	1340	26,453	1.455	1030	21,001	1.747	510	10,224	1.584

Own elaboration made with data provided by Andalusian Regional Government (Almería, Spain) [17,30].

**Table A2:** Revenues and expenses per hectare imputed to the papaya cultivation in a 30-month cycle (2016–2018).

Papaya Cultivation	Papaya	Papaya	Papaya	Papaya	Papaya	Total
	February 2016 to July 2016	August 2016 to January 2017	February 2017 to July 2017	August 2017 to January 2018	February 2018 to July 2018	February 2016 to July 2018
Total annual revenues (€)	-	16,874	130,268	67,523	33,347	248,013
Yield (kg·ha^−1^)	-	12,532	127,809	85,727	50,213	276,282
Average price (€·kg^−1^)		1.326	1.013	0.784	0.665	0.893
Total variable cost (€)	42,022	26,162	27,645	19,889	19,900	135,618
Technical assessment (€)	152	152	155	155	158	772
Soil preparation (€)	3839	-	-	-	-	3839
Covering and structure (€)	2443	2443	2444	2480	2487	12,297
Seeds and seedling production (€)	13,500	-	-	-	-	13,500
Growing and development until 1^st^ inflorescence (€)	22,087	11,044	-	-	-	33,131
Flowering periods until 1^st^ harvesting season (€)	-	12,523	25,047	-	-	37,570
From the 1^st^ harvesting season until the end of the cultivation (€)	-	-	-	17,255	17,255	34,510
Total fixed costs (€)	10,912	10,912	10,996	10,912	11,127	54,859
Soil maintenance (€)	1014	1014	1029	1014	1053	5125
Covering and structure (€)	2030	2030	2060	2030	2108	10,257
Energy and fixed supplies (€)	802	802	814	802	832	4051
Insurance, management, and financial services (€)	1765	1765	1792	1765	1833	8920
Equipment and irrigation system (€)	5301	5301	5301	5301	5301	26,506
Total cost (€)	52,934	37,074	38,641	30,801	31,027	190,477
Net profit before taxes (€)	−52,934	−20,201	91,627	36,722	2320	57,535

Own elaboration.

**Table A3:** Revenues and expenses per hectare imputed to the cultivation of the horticultural alternative 1 in a 30-month cycle (2016–2018).

Horticultural Alternative 1	Watermelon	Tomato	Courgette	Sweet Pepper	Watermelon	Total
	February 2016 to July 2016	August 2016 to January 2017	February 2017 to July 2017	August 2017 to January 2018	February 2018 to July 2018	February 2016 to July 2018
Total annual revenues (€)	19,716	53,385	38,340	61,199	21,164	193,805
Yield (kg·ha^−1^)	62,000	101,300	56,300	67,400	52,000	
Average price (€·kg^−1^)	0.318	0.527	0.681	0.908	0.407	
Total variable cost (€)	18,532	52,092	36,406	51,117	19,243	177,390
Technical assessment (€)	152	152	155	155	158	772
Soil preparation (€)	3839	3839	3897	3897	3986	19,458
Covering and structure (€)	2337	2337	2372	2372	2427	11,846
Seeds and seedling production (€)	1993	5639	1277	7005	2070	17,983
Growing and development until 1^st^ inflorescence (€)	3173	7385	14,942	13,151	3295	41,947
Flowering periods until 1^st^ harvesting season (€)	-	15,573	-	10,206	-	25,779
From the 1^st^ harvesting season until the end of the cultivation (€)	7037	17,166	13,763	14,332	7307	59,605
Total fixed costs (€)	9678	9678	9823	9823	10,049	49,052
Soil maintenance (€)	1014	1014	1029	1029	1053	5140
Covering and structure (€)	2030	2030	2060	2060	2108	10,287
Energy and fixed supplies (€)	802	802	814	814	832	4063
Insurance, management, and financial services (€)	1765	1765	1792	1792	1833	8947
Equipment and irrigation system (€)	4067	4067	4128	4128	4223	20,615
Total cost (€)	28,211	61,770	46,230	60,940	29,292	226,443
Net profit before taxes (€)	−8495	−8385	−7889	259	−8128	−32,638

Own elaboration made with revenue data provided by Andalusian Regional Government [17,30].

**Table A4:** Revenues and expenses per hectare imputed to the cultivation of the horticultural alternative 2 in a 30-month cycle (2016–2018).

Horticultural Alternative 2	Tomato	Cucumber	Aubergine	Green Bean	Melon	Total
	February 2016 to July 2016	August 2016 to January 2017	February 2017 to July 2017	August 2017 to January 2018	February 2018 to July 2018	February 2016 to July 2018
Total annual revenues (€)	53,385	36,491	56,930	35,639	21,400	203,845
Yield (kg·ha^−1^)	101,300	87,300	78,200	20,400	40,000	
Average price (€·kg^−1^)	0.527	0.418	0.728	1.747	0.535	
Total variable cost (€)	52,092	42,301	41,681	25,851	29,318	191,244
Technical assessment (€)	152	152	155	155	158	772
Soil preparation (€)	3839	3839	3897	3897	3986	19,458
Covering and structure (€)	2337	2337	2372	2372	2427	11,846
Seeds and seedling production (€)	5639	6901	2984	1470	5594	22,588
Growing and development until 1^st^ inflorescence (€)	7385	8472	6907	6889	5748	35,401
Flowering periods until 1^st^ harvesting season (€)	15,573	7063	8694	2349	-	33,679
From the 1^st^ harvesting season until the end of the cultivation (€)	17,166	13,536	16,673	8720	11,404	67,499
Total fixed costs (€)	9678	9678	9823	9823	10,049	49,052
Soil maintenance (€)	1014	1014	1029	1029	1053	5140
Covering and structure (€)	2030	2030	2060	2060	2108	10,287
Energy and fixed supplies (€)	802	802	814	814	832	4063
Insurance, management, and financial services (€)	1765	1765	1792	1792	1833	8947
Equipment and irrigation system (€)	4067	4067	4128	4128	4223	20,615
Total cost (€)	61,770	51,980	51,505	35,674	39,367	240,296
Net profit before taxes (€)	−8385	−15,488	5425	−36	−17,967	−36,451

Own elaboration made with revenue data provided by Andalusian Regional Government [17,30].

**Table A5:** Revenues and expenses per hectare imputed to the cultivation of the horticultural alternative 3 in a 30-month cycle (2016–2018).

Horticultural Alternative 3	Melon	Sweet Pepper	Watermelon	Tomato	Melon	Total
	February 2016 to July 2016	August 2016 to January 2017	February 2017 to July 2017	August 2017 to January 2018	February 2018 to July 2018	February 2016 to July 2018
Total annual revenues (€)	18,377	57,845	20,405	70,472	21,400	188,498
Yield (kg·ha^−1^)	39,100	70,200	62,400	98,700	40,000	
Average price (€·kg^−1^)	0.470	0.824	0.327	0.714	0.535	
Total variable cost (€)	28,235	50,361	18,810	52,873	29,318	179,599
Technical assessment (€)	152	152	155	155	158	772
Soil preparation (€)	3839	3839	3897	3897	3986	19,458
Covering and structure (€)	2337	2337	2372	2372	2427	11,846
Seeds and seedling production (€)	5388	6901	2023	5723	5594	25,630
Growing and development until 1^st^ inflorescence (€)	5536	12,957	3221	7496	5748	34,958
Flowering periods until 1^st^ harvesting season (€)	-	10,055	-	15,807	-	25,862
From the 1^st^ harvesting season until the end of the cultivation (€)	10,983	14,120	7143	17,424	11,404	61,073
Total fixed costs (€)	9678	9678	9823	9823	10,049	49,052
Soil maintenance (€)	1014	1014	1029	1029	1053	5140
Covering and structure (€)	2030	2030	2060	2060	2108	10,287
Energy and fixed supplies (€)	802	802	814	814	832	4063
Insurance, management, and financial services (€)	1765	1765	1792	1792	1833	8947
Equipment and irrigation system (€)	4067	4067	4128	4128	4223	20,615
Total cost (€)	37,914	60,040	28,634	62,697	39,367	228,651
Net profit before taxes (€)	−19,537	−2195	−8229	7775	−17,967	−40,153

Own elaboration made with revenue data provided by Andalusian Regional Government [17,30].

**Table A6:** Revenues and expenses per hectare imputed to the cultivation of the horticultural alternative 4 in a 30-month cycle (2016–2018).

Horticultural Alternative 4	Courgette	Aubergine	Melon	Sweet Peper	Watermelon	Total
	February 2016 to July 2016	August 2016 to January 2017	February 2017 to July 2017	August 2017 to January 2018	February 2018 to July 2018	February 2016 to July 2018
Total annual revenues (€)	27,824	29,717	18,861	61,199	21,164	158,765
Yield (kg·ha^−1^)	56,900	80,100	42,100	67,400	52,000	
Average price (€·kg^−1^)	0.489	0.371	0.448	0.908	0.407	
Total variable cost (€)	35,868	41,065	28,659	51,117	19,243	175,953
Technical assessment (€)	152	152	155	155	158	772
Soil preparation (€)	3839	3839	3897	3897	3986	19,458
Covering and structure (€)	2337	2337	2372	2372	2427	11,846
Seeds and seedling production (€)	1258	2940	5469	7005	2070	18,741
Growing and development until 1^st^ inflorescence (€)	14,722	6805	5619	13,151	3295	43,591
Flowering periods until 1^st^ harvesting season (€)	-	8565	-	10,206	-	18,771
From the 1^st^ harvesting season until the end of the cultivation (€)	13,560	16,427	11,148	14,332	7307	62,772
Total fixed costs (€)	9678	9678	9823	9823	10,049	49,052
Soil maintenance (€)	1014	1014	1029	1029	1053	5140
Covering and structure (€)	2030	2030	2060	2060	2108	10,287
Energy and fixed supplies (€)	802	802	814	814	832	4063
Insurance, management, and financial services (€)	1765	1765	1792	1792	1833	8947
Equipment and irrigation system (€)	4067	4067	4128	4128	4223	20,615
Total cost (€)	45,546	50,744	38,482	60,940	29,292	225,005
Net profit before taxes (€)	−17,722	−21,026	−19,622	259	−8128	−66,240

Own elaboration made with revenue data provided by Andalusian Regional Government [17,30].

**Table A7:** Revenues and expenses per hectare imputed to the cultivation of the horticultural alternative 5 in a 30-month cycle (2016–2018).

Horticultural Alternative 5	Courgette	Tomato	Tomato	Tomato	Tomato	Total
	February 2016 to July 2016	August 2016 to January 2017	February 2017 to July 2017	August 2017 to January 2018	February 2018 to July 2018	February 2016 to July 2018
Total annual revenues (€)	27,824	48,895	73,343	52,599	78,899	281,560
Yield (kg·ha^−1^)	56,900	78,800	118,200	78,800	118,200	
Average price (€·kg^−1^)	0.489	0.621	0.621	0.668	0.668	
Total variable cost (€)	35,868	45,977	30,156	46,755	36,214	194,971
Technical assessment (€)	152	152	155	155	158	772
Soil preparation (€)	3839	3839	-	3897	-	11,575
Covering and structure (€)	2337	2337	2372	2372	2427	11,846
Seeds and seedling production (€)	1258	5638	-	5723	2070	14,689
Growing and development until 1^st^ inflorescence (€)	14,722	7385	-	7496	3295	32,898
Flowering periods until 1^st^ harvesting season (€)	-	15,573	-	15,807	-	31,380
From the 1^st^ harvesting season until the end of the cultivation (€)	13,560	11,052	27,629	11,306	28,265	91,811
Total fixed costs (€)	10,662	10,662	10,746	10,746	10,877	53,693
Soil maintenance (€)	1014	1014	1029	1029	1053	5140
Covering and structure (€)	2030	2030	2060	2060	2108	10,287
Energy and fixed supplies (€)	802	802	814	814	832	4063
Insurance, management, and financial services (€)	1765	1765	1792	1792	1833	8947
Equipment and irrigation system (€)	5051	5051	5051	5051	5051	25,256
Total cost (€)	46,530	56,639	40,902	57,501	47,092	248,664
Net profit before taxes (€)	−18,706	−7743	32,441	−4902	31,807	32,896

Own elaboration made with revenue data provided by the Andalusian Regional Government [17,30].
